# QTL analysis of important agronomic traits and metabolites in foxtail millet (*Setaria italica*) by RIL population and widely targeted metabolome

**DOI:** 10.3389/fpls.2022.1035906

**Published:** 2023-01-10

**Authors:** Wei Wei, Shuangdong Li, Peiyu Li, Kuohai Yu, Guangyu Fan, Yixiang Wang, Fang Zhao, Xiaolei Zhang, Xiaolei Feng, Gaolei Shi, Weiqin Zhang, Guoliang Song, Wenhan Dan, Feng Wang, Yali Zhang, Xinru Li, Dequan Wang, Wenying Zhang, Jingjing Pei, Xiaoming Wang, Zhihai Zhao

**Affiliations:** ^1^ Institute of Millet, Zhangjiakou Academy of Agricultural Science, Zhangjiakou, China; ^2^ Wuhan Metware Biotechnology Co., Ltd., Wuhan, China

**Keywords:** foxtail millet, RIL population, widely targeted metabolomics, metabolic QTL, phenotypic QTL

## Abstract

As a bridge between genome and phenotype, metabolome is closely related to plant growth and development. However, the research on the combination of genome, metabolome and multiple agronomic traits in foxtail millet (*Setaria italica*) is insufficient. Here, based on the linkage analysis of 3,452 metabolites *via* with high-quality genetic linkage maps, we detected a total of 1,049 metabolic quantitative trait loci (mQTLs) distributed in 11 hotspots, and 28 metabolite-related candidate genes were mined from 14 mQTLs. In addition, 136 single-environment phenotypic QTL (pQTLs) related to 63 phenotypes were identified by linkage analysis, and there were 12 hotspots on these pQTLs. We futher dissected 39 candidate genes related to agronomic traits through metabolite-phenotype correlation and gene function analysis, including *Sd1* semidwarf gene, which can affect plant height by regulating GA synthesis. Combined correlation network and QTL analysis, we found that flavonoid-lignin pathway maybe closely related to plant architecture and yield in foxtail millet. For example, the correlation coefficient between apigenin 7-rutinoside and stem diameter reached 0.98, and they were co-located at 41.33-44.15 Mb of chromosome 5, further gene function analysis revealed that 5 flavonoid pathway genes, as well as *Sd1*, were located in this interval . Therefore, the correlation and co-localization between flavonoid-lignins and plant architecture may be due to the close linkage of their regulatory genes in millet. Besides, we also found that a combination of genomic and metabolomic for BLUP analysis can better predict plant agronomic traits than genomic or metabolomic data, independently. In conclusion, the combined analysis of mQTL and pQTL in millet have linked genetic, metabolic and agronomic traits, and is of great significance for metabolite-related molecular assisted breeding.

## 1 Introduction

Foxtail millet (*Setaria italica*) is one of the earliest domesticated crops from the green foxtail (*Setaria viridis*) (Bennetzen et al., 2012; Pant et al., 2016; Yang et al., 2022). Millet has the characteristics of wide adaptability and yield stability, and it adapt to high temperature and arid environment well (Yuan et al., 2021; Han et al., 2022). In addition to its high nutritional value (Li et al., 2018; Ma et al., 2022), millet is also an ideal model crop for studying grass crops due to its small genome and close relatedness to many important grass crops, such as switchgrass (*Panicum virgatum*), napiergrass(*Pennisetum purpureum*), pearl millet (Li et al., 2022; Xing et al., 2022). Therefore, the research on the formation of millet phenotype, genetic mechanism and molecular breeding is of great significance.

Linkage-based quantitative trait loci (QTL) mapping had been conducted in millet for several agronomic traits including yield, grain weight, flowering days and seed number (Jaiswal et al., 2019). Ni et al. performed QTL mapping of nine agronomic traits using the recombinant inbred line (RIL) population, five of which were controlled by single gene. They identified two QTLs for plant height, and a candidate gene showed 89% identity to the known rice gibberellin-synthesis gene *Sd1* (Ni et al., 2017). Therefore, QTL is an effective method for agronomic traits analysis, as well as metabolites content. Metabolome is a powerful tool to systematically explore genotype-phenotype relationships in plants, the level of metabolites detected in plants further reflects the growth status (Fang et al., 2019). Combining mQTL with pQTL analysis can give a fuller picture of the molecular mechanisms of important traits from genomic and metabolic pathway.

With the gradual maturity of widely targeted metabolome detection technology, it is possible to detect and identify more than thousand metabolites at the same time (Chen et al., 2013). Thus, multi-omics integrated analysis including metabolome has become a research hotspot. In wheat, the genetic mechanism of metabolites was analyzed by mQTL, *in vitro* and it was confirmed that *TraesCS5D01G028100* and *TraesCS2B01G459900* have glycosyltransferase activities *in vitro*, which determined the accumulations in Apigenin and Trincin glycoside, respectively (Shi et al., 2020). The genetic analysis of rice combining metabolic profiling with an ultrahigh-density genetic map proved that a large number of mQTL can greatly accelerate the identification of gene functions, and advance the research on the genetic and biochemical basis of the metabolome (Gong et al., 2013). QTL analysis in metabolics have been carried out in several major crops and model plant species, including rice, wheat, tomato, and Arabidopsis (Gong et al., 2013; Knoch et al., 2017; Garbowicz et al., 2018; Shi et al., 2020), but few have been reported in millet.

Previous studies have found that metabolites can be used as biomarkers to predict complex agronomic traits, which could speed up the breeding process while reducing costs. Riedelsheimer used 285 inbred lines (285 × 2 = 570 hybrids) crossed with two subjects to predict traits in hybrid maize by RR-BLUP and found that the average predictability of seven traits ranged from 0.72 to 0.81 for SNPs and 0.60 to 0.80 for metabolites (Riedelsheimer et al., 2012). Xu et al. using metabolomic data from 210 RILs to predict thousand-grain weight (KGW) and other traits, found that LASSO and BLUP were the most effective methods for yield prediction, and nearly doubled the predictability when using metabolomic data compared to genomic data (Xu et al., 2016). The above studies suggest that metabolites are useful predictors for quantitative traits. However, the analysis of the association between metabolomics and phenotypes in millet has not yet been implemented.

In order to provide a more comprehensive understanding of the metabolites in millet, explore the genetic relationship between the metabolites and agronomic traits, we conducted metabolites and agronomic traits analysis in a RIL populations of 179 millet. Metabolites and agronomic traits were linkage analyzed using high-density genetic linkage maps to find QTLs, hotspots and co-localized locus. Besides, we found it was reliable that combining metabolomic data and genomic variation information can more effectively predict agronomic traits in millet, which increased our understanding of the relationship of metabolome-phenotype, and provides novel ideas for the selection and improvement of millet.

## 2 Materials and methods

### 2.1 Plant materials

The 179 lines from the RIL population developed from a cross between A2 (PTGMS A2) and Zhangzagu No.3 (Line 1484-5 × A2) was used in this study. The population was evaluated in natural field conditions in the experimental farm of Zhangjiakou Academy of Agricultural Science, Zhangjiakou, China (144°88’N, 40°77’E). A randomized block design was used during 2016-2018 cropping seasons. Each line was replicated three times (individual plants) and planted in a two-row plot of 1.5 m length with the spacing of 0.1 m between plants and 0.2 m between rows. Field management, including irrigation, fertilization, weeding and pest control, followed the standard agricultural practices in millet production. For each line, leaves from three plants were taken at the five-leaf stage and pooled for one biological replicate. Leaf samples for each line were selected for DNA or chemical extraction. All the samples were harvested at 10:00-12:00 on that day, placed in liquid N_2_ immediately and stored at -70°C until vacuum freeze-drying.

The 179 accessions used in this study were characterized by whole genome re-sequencing. DNA was isolated from young leaves using the CTAB method and sequencing libraries with short inserts were constructed following manufacturer’s instructions (Illumina). The samples were sequenced on an Illumina HiSeq 4000 platform. To retain reads of high quality, reads with fewer than 5% N (missing) bases and with fewer than 50% of bases of base quality < 5 were deemed as cleaned reads. All other reads were discarded.

### 2.2 Chemicals

All the chemicals were of analytical reagent grade. Gradient-grade methanol, acetonitrile and acetic acid were purchased from Merck Company, Germany (http://www.merck-chemicals.com). The water was doubly deionised with a Milli-Q water purification system (Millipore, Bedford, MA). Authentic standards were purchased from ANPEL, Shanghai, China (www.anpel.com.cn/cnw), BioBioPha Co., Ltd. (http://www.biobiopha.com/), and Sigma-Aldrich, USA (http://www.sigmaaldrich.com). Standards stock solutions were prepared using water, methanol and/or dimethyl sulfoxide (DMSO) as the solvent and stored at -20°C. Combined standard solutions of chemicals were prepared just before use by mixing individual stock solutions and diluting these mixtures with 70% aqueous methanol.

### 2.3 Sample preparation and extraction

The freeze-dried leaves were crushed using a mixer mill (MM 400, Retsch) with a zirconia beads for 1.5 min at 30 Hz. A 100 mg mass of powder was weighted and extracted overnight at 4°C with 1.0 ml of 70% aqueous methanol. Following centrifugation at 10, 000 g for 10 min, the extracts were filtered (SCAA-104, 0.22 μm pore size; ANPEL, Shanghai, China, http://www.anpel.com.cn/) before liquid chromatography-mass spectrometry (LC-MS) analysis.

### 2.4 LC-MS conditions

The sample extracts were analyzed using an LC-ESI-MS/MS system (HPLC, Shim-pack UFLC SHIMADZU CBM30A system, www.shimadzu.com.cn/; MS, Applied Biosystems 6500 Q TRAP, www.appliedbiosystems.com.cn/). The analytical conditions were as follows, HPLC: column, Waters ACQUITY UPLC HSS T3 C18 (1.8 µm, 2.1 mm*100 mm); solvent system, water (0.04% acetic acid): acetonitrile (0.04% acetic acid); gradient program, 100:0 V/V at 0 min, 5:95V/V at 10.0 min, 5:95V/V at 11.0 min, 95:5 V/V at 11.1 min, 95:5 V/V at 15.0 min; flow rate, 0.35 ml/min; temperature, 40°C; and injection volume: 5 μl. The effluent was alternatively connected to an ESI-triple quadrupole-linear ion trap (Q TRAP)-MS.

LIT and triple quadrupole (QQQ) scans were acquired on a triple quadrupole-linear ion trap mass spectrometer (Q TRAP) using an API 6500 Q TRAP LC/MS/MS System, which was equipped with an ESI Turbo Ion-Spray interface operated in a positive ion mode and controlled by Analyst 1.6.3 software (AB Sciex). The ESI source operation parameters were as follows: ion source, turbo spray; source temperature 550°C; ion spray voltage (IS) 5,500 V; ion source gas I (GSI), gas II (GSII), curtain gas (CUR) were set at 55, 60, and 30.0 psi, respectively; and the collision gas (CAD) was high. Instrument tuning and mass calibration were performed with 10 and 100 μmol/L polypropylene glycol solutions in QQQ and LIT modes, respectively. The QQQ scans were acquired as MRM experiments with the collision gas (nitrogen) set to 5 psi. The DP and CE for individual MRM transitions were performed with further DP and CE optimization. A specific set of MRM transitions was monitored for each period according to the metabolites that were eluted within this period.

### 2.5 Statistical analysis

The metabolite data were log2-transformed for statistical analysis to improve normality. Broad-sense heritability (H^2^) was calculated using the following formula: H^2 =^ 1-10^-2LOD/^ *^n^*, where *n* is the sample size (Arends et al., 2010). The values of the coefficient of variation (CV) were calculated for each metabolite and agronomic trait (three-year data are calculated separately) expressed as S/A, where S and A represent the standard deviation and the average of metabolite and agronomic trait in the population, respectively. Pearson’s correlation and the statistical significance between traits were estimated using programs housed in R (http://www.r-project.org/). Visualization correlation networks were constructed using Cytoscape 3.7.0 (Smoot et al., 2010).

### 2.6 QTL mapping and hotspot identification

A high-density genetic map was constructed for the RILs (Zhang et al., 2017). The QTL analysis of each trait was performed using the R package qtl version 1.46.2 (https://rqtl.org/), with a scanning step of 0.1 cM and PIN (probability in stepwise regression) of 0.01 (Li et al., 2007). The LOD threshold was set to 2.5 for both metabolites and agronomic traits. The confidence interval for each QTL was assigned as a 1.5-LOD drop of the peak. The additive effect and percentage of phenotypic variance associated with a QTL (contribution) were estimated using the same program. For metabolic QTL (mQTL), the QTL intervals of the same metabolite overlapped in two replicates, will be selected for research. If the phenotypic variance was greater than 15%, it was considered a major QTL (Salvi and Tuberosa, 2005).

The whole genome was divided into 3 Mb partitions, and the number of mQTL per partition was counted. Using 1,000 permutation tests, each mQTL was randomly assigned to a 3 Mb interval, and the number of mQTLs obtained in each interval was counted. The cut-off number of mQTLs per 3 Mb by chance alone was 14 in mature seeds with P < 0.05, respectively. A larger number was regarded as a mQTL hotspot (Gong et al., 2013).

### 2.7 Phylogenetic analysis

The amino acid sequences of reported genes were obtained from NCBI according to their accession numbers (http://www.ncbi.nlm.nih.gov/). Candidate gene information in this study was obtained from the draft assembly of the millet genome (Bennetzen et al., 2012). The alignment of amino acid sequences was performed using ClustalW bundled in MEGA 5, and neighbor-joining trees were constructed using MEGA 5 software with all default parameters. The reliability of the reconstructed tree was evaluated using a bootstrap test with 1000 replicates.

### 2.8 Prediction of agronomic traits

A total of 63 agronomic traits were determined in 2016-2018 (Zhangjiakou) and the detailed information were shown in the **Supplementary table 1**. Most phenotypes were measured using common methods such as plant height, thousand-grain weight and so on (Zhang et al., 2017; Fan et al., 2019). Other traits, such as anther color, which was observed at the time of floret dehiscence at anthesis, was scored on a scale of 1 point for white, 3 points for yellow and 5 points for brown; spike neck shape was to observe the bending degree and posture of stem node under spike, the morphology of these traits were evaluated and scored by experienced personnel. Briefly, genomic (2,202 bins of 33,579 SNPs integrated), metabolomic (3,452 metabolic signals) and multi-omics data (genomic and metabolomic data integration) were used to predict 63 agronomic traits using the BLUP method in R (rrBLUP) (Endelman, 2011) and LASSO methods in R (glmnet) (Hastie et al., 2017). The predictability was measured using a 10-fold cross-validation method. The 179 RILs were then randomly divided into 10 groups, 9 of which were used to construct the model. The remaining RILs were predicted. The predictive power (predictability) is defined as the Pearson’s correlation coefficient between the phenotypic observations and the predicted values (Shi et al., 2020).

## 3 Results

### 3.1 Metabolome profiling of leaf tissues from foxtail millet RIL population

**Figure 1:**
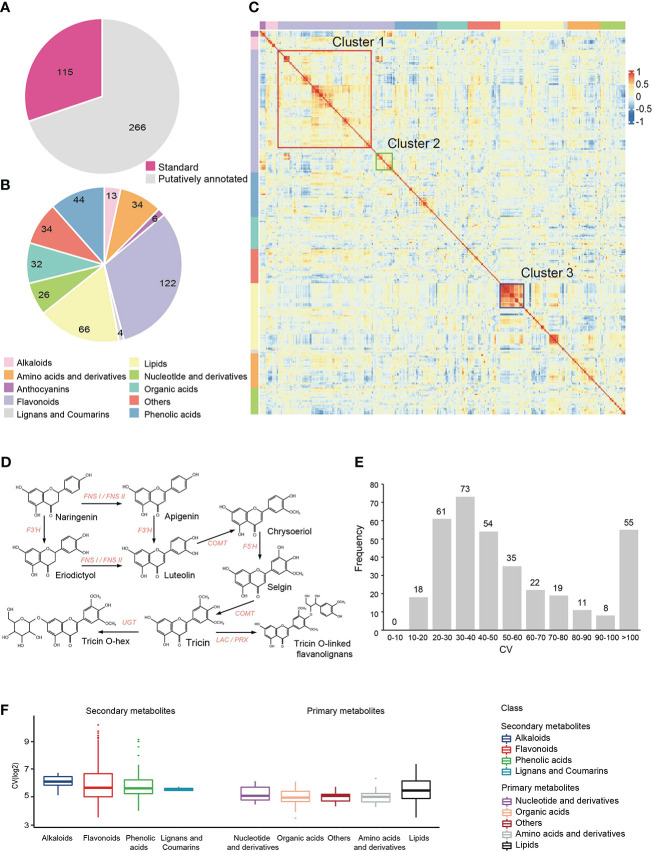
Metabolic profiling in millet RIL population. **(A, B)** Number of detected metabolites and their classification. **(C)** Cluster heatmap of correlations among annotated metabolites. Pairwise Pearson’s correlations are shown in a heatmap, metabolites in same class are sorted according to correlation-based hierarchical cluster analysis. The level of correlation is indicated by red (positive correlation) and blue (negative correlation). **(D)** Metabolic pathways of flavonoids in millet (Cluster 1). **(E)** Distribution of the coefficients of variation (CV) of annoted metabolic traits in the millet RIL population. **(F)** Statistical analysis of the coefficient of variation (CV) of each class of metabolites in the millet RIL population.

Using widely targeted liquid chromatography-tandem mass spectrometry (LC-MS/MS)-based metabolic profiling method (Chen et al., 2013), we performed metabolic profiling with leaves at the five-leaf stage from 179 accessions derived from a cross between two elite foxtail millet varieties, A2 and Zhangzagu No.3. A total of 3,452 reproducible metabolite signals were detected, of which 381 metabolites were qualitatively analyzed through standard comparison and putatively annotated (**Figure 1A**). These annotated metabolites achieved a coverage of multiple important metabolic pathways for plants, including Flavonoids, Lipids, Phenolic acids, Amino acids and derivatives, Organic acids, Nucleotide and derivatives, Alkaloids, Anthocyanins, Lignans and Coumarins (**Figure 1B**, **Supplementary Table 2**).

Pearson’s correlation coefficient of 381 annotated metabolites in 179 millets were calculated to explore the links between different metabolic pathways (**Figure 1C**, **Supplementary Table 3**). We found that the number of metabolites with significant positive correlation (r > 0.5, p < 0.05, shown in red) were far more than those with significant negative correlation (r < -0.5, p < 0.05, shown in blue). We also found some closely related areas in correlation heatmap, especially for lipid metabolites, which indicated that these lipid metabolic pathways were relatively independent (Cluster 3, shown in blue box) of other types of metabolites. It is worth noting that flavonoids has formed two different clusters (Cluster 1, Cluster 2), and the two clusters showed significant negative correlation with each other. Cluster 1 (shown in red box) contained more flavonoid metabolites mainly derived from the Apigenin - Luteolin - Chrysoeriol - Selgin - Tricin- flavonoid lignin pathway (**Figure 1D**), while Cluster 2 (shown in green box) was mainly a group of flavonoids with the same modification that might have a relationship of metabolic substrate competition.

Coefficient of variation (CV) is often used to assess the extent of metabolic differences between populations. There were 76.64% annotated metabolites with a CV above 30%, (**Figure 1E**, **Supplementary Table 2**), which suggested great variations of metabolites in different foxtail millet varieties. Moreover, the CV of primary metabolites were lower (*t*-test, *p*-value=1.63E-7) than that of secondary metabolites (**Figure 1F**, **Supplementary Table 4**), indicating that secondary metabolites have greater variation than primary metabolites. Generally, secondary metabolites with greater variation in the population have important functions during plant growth and facilitate the mining of related genetic factors, such as anthocyanins and flavonoids, whose CV were 413.03% and 125.91%, respectively

### 3.2 mQTL analysis of foxtail millet

The high-density genetic linkage map for “Zhangzagu No.03 × A2” RIL population used in this study was constructed as previously (Zhang et al., 2017). The linkage map consisted of 2,202 bin and 33,579 SNPs from all 9 chromosomes of foxtail millet. The genetic map spanned 1,934.6 cM of the foxtail millet genome, with average 0.96 cM per bin. Based on the high-density genetic linkage map, a total of 1,049 mQTLs (LOD > 2.5) from 992 metabolites were mapped, among which 114 annotated metabolites were mapped to 130 mQTLs (**Supplementary Table 5**). According to the mapping results of mQTL, the proportion of mQTLs identified by secondary metabolites was higher than that in primary metabolites (**Supplementary Figures 1A, B**).

**Figure 2:**
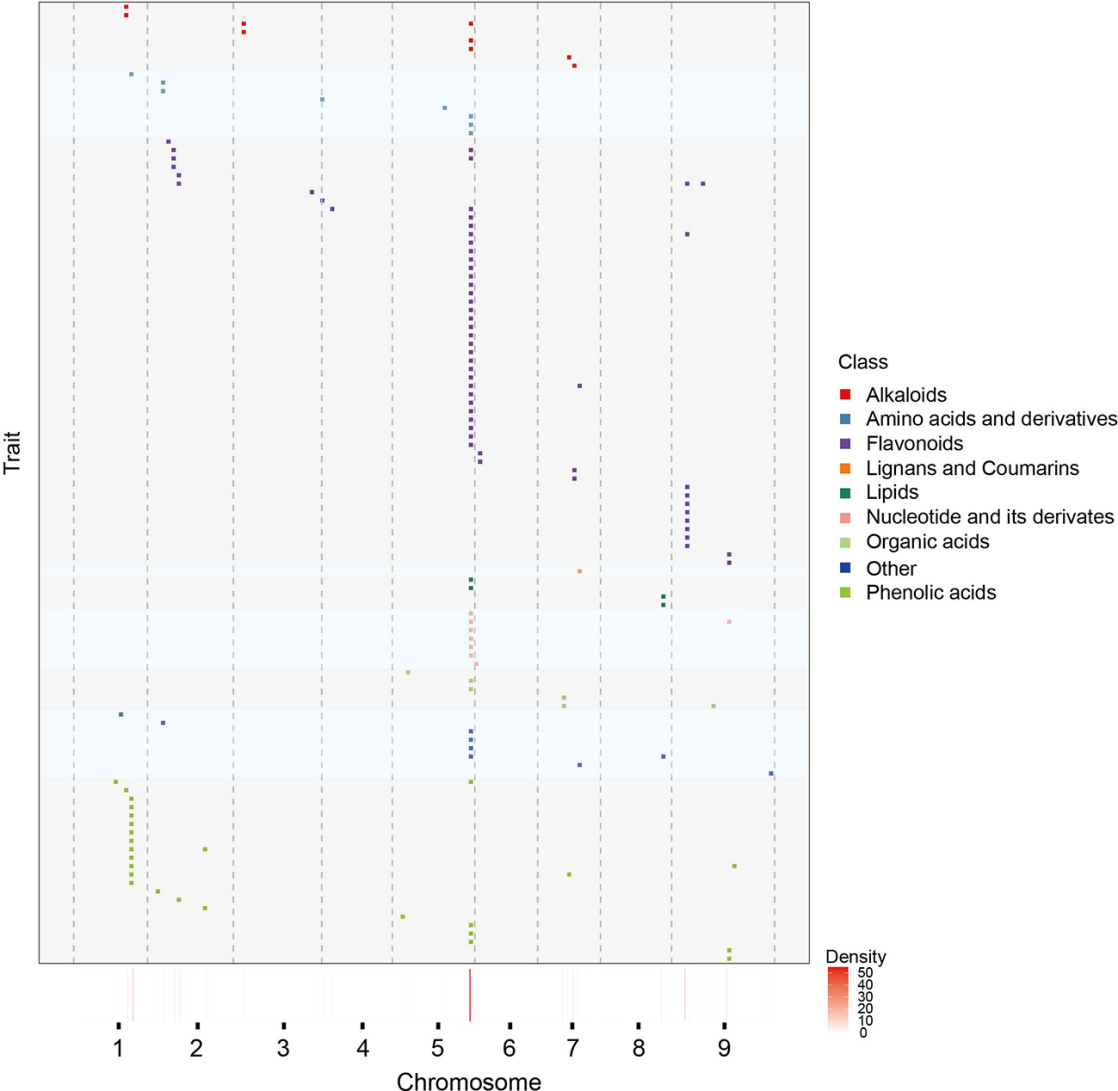
Chromosomal distribution of mQTLs identicfied from annotated metabolites. Distribution of mQTLs of 467 known metabolites on chromosomes. Each row represents the QTL mapping of single metabolic traits. Metabolites from different class are marked by distinct colours. The x-axis indicates the positions across the millet genome. The heatmap under the x-axis illustrates the density of QTL across the genome. The window size is 3 Mb.

To have a more detailed understanding of mQTL results, we have also analyzed the distribution of mQTLs, a total of 11 mQTL hotspots on seven chromosomes were identified, indicating that some regulated genes of multiple metabolites may located in these regions (**Figure 2**). It’s worth noting that there were 59 mQTLs from 55 annotated metabolites co-localized on the hotspot_5 (**Supplementary Table 5, 6**). Most of these metabolites were involved in the phenylpropane and alkaloid-putrescine metabolic pathway, thus we believed that there may be regulatory genes of multiple metabolic pathways in hotspot_5. However, fewer mQTLs were detected on other chromosomes than expected, especially on chromosome 4 (**Supplementary Table 7**).

### 3.3 Unearthing of mQTL potential candidate genes

Stable high-quality mQTLs can help us to discover potential candidate genes. Based on the structural characteristics of the metabolites and metabolic pathways have been reported, we screened 28 metabolite-related candidate genes from 14 mQTLs (**Table 1**). The most significant mQTL for GZ2498 (Chrysoeriol-O-hexoside-malonyl-hexoside) was a segment on chromosome 3 in 49.35 Mb (241.3-251.4 cM, LOD = 3.18, PVE = 6.44%). As a candidate gene at this locus, *Si021852m* had high homology to the reported Arabidopsis gene *AtDTX41* (Blastp E-value=0.0, Identity: 60.9%), which encodes a MATE efflux family protein involved in the pathway flavonoid biosynthesis (Zhao and Dixon, 2009). QTL of GZ2770 (Tricin O-sinapoylhexoside) was mapped to the 2.44-3.98 Mb on Chromosome 9 (28.3-45.8 cM, LOD=2.68 and PVE=6.44), *Si006338m*, *Si008637m* and *Si006185m* in the interval were homologous with a anthocyanidin 3-O-glucosyltransferase gene *ZmBZ1* (Blastp E-value=0.0, Identity: 81.9%) in maize, a flavonoid 3-dioxygenase 3 gene *OsF3’H-3* (Blastp E-value=4.4E-64, Identity: 38.0%) in rice and a quercetin 3-O-glucosltransferse gene *AtUGT73B4* (Blastp E-value=0.0, Identity: 67.0%) in Arabidopsis, respectively (Roth et al., 1991; Lim et al., 2004; Kim et al., 2008; Byeon and Back, 2015). Besides, at the QTL of GZ2525 (apigenin-C-rutinoside) in 21.00-36.05 Mb on Chr 9 (112.6-132.7 cM, LOD=2.74 and PVE=4.02), we screened two genes *Si035224m* and *Si035935m*, they are highly homologous with the *OsF3’H* gene (*Si035224m*: Blastp E-value=0.0, Identity: 81.1%; *Si035935m*: Blastp E-value=0.0, Identity: 80.6%). In Rice, *OsF3’H* had been reported as flavonoid 3-hydroxylase which catalyzes the 3’-hydroxylation of the flavonoid B-ring to the 3’,4’-hydroxylated state (Lam et al., 2015). These all demonstrated the high quality and reliability of the mQTL of this study.

**Table 1:** Candidate genes based on mQTL results.

Trait ID	Compound_name	Class	Chr	LOD	PVE	Interval (Mb)	Gene ID	Description
GZ0329	L-Phenylalanine	Amino acids	1	3.27	10.30	29.64-31.51	Si016926m	HCT
GZ0789	p-Coumaric acid	Phenolic acids	1	4.03	2.25	30.72-32.10	Si016504m;Si016467m	PAL
							Si019385m;Si016478m	PAL
							Si016475m	PAL
GZ2674	Tricin O-feruloylhexoside O-hexoside	Flavonoids	2	4.25	7.31	9.74-15.77	Si029576m;Si029633m	UDP-RhaT
GZ2498	chrysoeriol-O-hexoside-malonyl-hexoside	Flavonoids	3	3.18	6.44	48.89-49.83	Si021852m	MATE
GZ2770	Tricin O-sinapoylhexoside	Flavonoids	4	2.68	6.11	2.44-3.98	Si006338m	Anthocyanidin 3-O GT
							Si007997m	UDP-GT
GZ0710	Luteolin C-hexoside	Flavonoids	5	3.17	6.81	41.98-43.61	Si001301m;Si001332m	UDP-RhaT
GZ0064	L-Serine	Amino acids	5	8.81	21.39	43.37-44.01	Si001325m;Si001577m	AAPS
GZ2434	luteolin-C-hexoside	Flavonoids	5	2.62	7.81	43.06-44.15	Si000845m	Lc
GZ1147	4-Methoxycinnamic acid	Phenolic acids	7	2.54	7.96	13.50-16.44	Si009584m	4CL
GZ2259	N-Feruloyl spermidine	Alkaloids	7	2.76	23.50	17.00-18.37	Si010179m	ACT
GZ2803	Chrysin 5-O-glucoside (Toringin)	Flavonoids	7	2.56	4.95	17.89-19.31	Si010039m	Anthocyanidin 3-O GT
GZ0609	Luteolin-C-pentosyl-C-hexoside	Flavonoids	9	4.03	8.53	6.30-8.61	Si040642m	F7,3GT
GZ0609	Luteolin-C-pentosyl-C-hexoside	Flavonoids	9	4.03	8.53	6.30-8.61	Si040087m;Si040021m	RhaT
							Si038926m;Si035772m	RhaT
GZ2525	apigenin-C-rutinoside	Flavonoids	9	2.75	4.02	14.33-19.47	Si039984m	FCGT
							Si035224m;Si035935m	F3’,4’H
							Si035595m	F5GT

**Figure 3:**
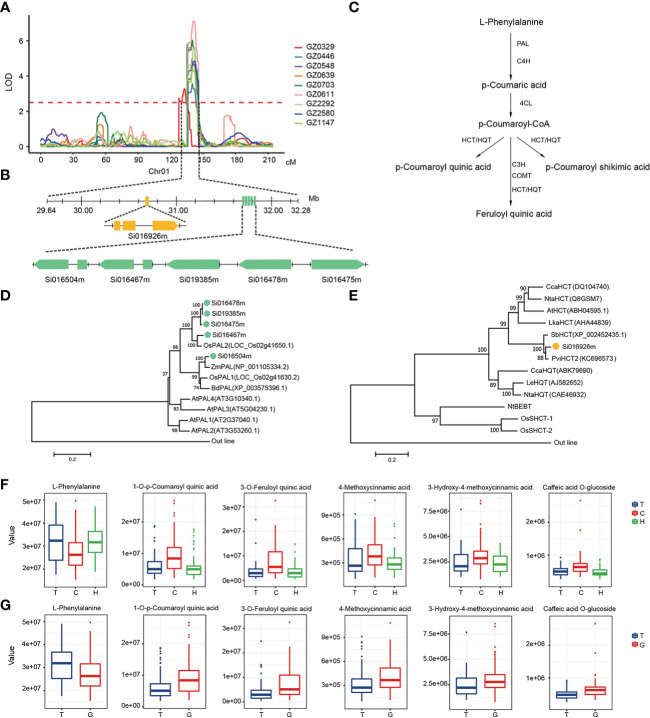
Candidate genes for a mQTL hotspot on chromosome 1 for phenylpropanoid metabolites. **(A)** LOD curves of QTL mapping of the phenylpropanoid metabolites accumulation on chromosome 1. **(B)** Gene model of candidate genes. The orange box represents the coding sequence of *HCT*, the green boxes represent the coding sequence of *PALs*. **(C)** Metabolic pathways of phenylpropanoid metabolites. **(D, E)** An unrooted phylogenetic tree of the candidate genes protein was constructed as described in Methods. Bootstrap values >70% (based on 1,000 replications) are indicated at each node (bar: 0.2 amino acid substitutions per site). **(F, G)** The effect of different alleles on the content of some phenylpropanoid metabolites. GZ0329, L-Phenylalanine; GZ0446, 3-O-p-coumaroyl quinic acid O-hexoside; GZ0548, 1-O-p-Coumaroyl quinic acid; GZ0639, 5-O-p-Coumaroyl quinic acid; GZ0703, 3-O-p-Coumaroyl quinic acid; GZ0611, Caffeic acid O-glucoside; GZ2292, 3-O-Feruloyl quinic acid; GZ2580, 3-Hydroxy-4-methoxycinnamic acid; GZ1147, 4-Methoxycinnamic acid.

We noticed that multiple phenolic acids at the upstream of the phenylpropane metabolic pathway were mapped to hotspot_2 with high LOD and PVE on chromosome 1 (**Figure 3A**). Through the annotation of metabolite pathway and gene function, we had unearthed 6 candidate genes in this locus, including a Hydroxycinnamoyltransferase (*HCT*) *Si016926m* and 5 redundant Phenylalanine ammonia-lyase (*PAL*) (**Figure 3B**). Orthologs of these candidate genes had been reported to play an important role in the regulation of phenolic acid synthesis in the phenylpropane metabolic pathway (**Figure 3C**). Phylogenetic analysis showed that the *PAL* genes were clustering with the reported *PAL* from monocotyledonous cereal crops, like rice and triticum, rather than the *PAL* from dicotyledonous plants such as Arabidopsis (Zhu et al., 1995; Liao et al., 1996) (**Figure 3D**). *Si016926m* clustering with the *HCT/HQT* genes that had been reported to transfer hydroxycinnamate to shikimate or quinine, and is involved in the formation of phenolic acid (Chao et al., 2021) (**Figure 3E**).

In order to further dissect the function variation of candidate genes, we analyzed the SNPs within the candidate genes. A total of 30 SNPs in these 6 candidate genes were related to at least one metabolite in the pathway (**Supplementary Table 8**). Among them, a non-synonymous mutation SNP (SNP1:3064324, C/T) in *Si016926m* caused the conversion between the basic amino acid lysine (Lys, K) and the neutral amino acid aspartyl (Asn, N). There were significant differences in metabolites content among different SNP haplotypes (p < 1E-03). As a upstream metabolite of phenylpropane metabolic pathway, GZ0329 (L-Phenylalanine) content in millet varieties with C is lower than those millet varieties with T or heterozygous in this site. On the contrary, the content of other downstream metabolites in millet with C had higher content than those millet varieties with T or heterozygous in the same SNP (**Figure 3F**). Moreover, a non-synonymous mutation SNP (SNP1:3176740, T/G) was found in the coding region of *Si016467m*, which can lead to the conversion between non-polar alanine (Ala, A) and polar threonine (Thr, T)The content of GZ0329 in the millet with T was significantly higher than that of the G, while other downstream substances such as GZ0548 (1-O-p-Coumaroyl quinic acid) and GZ2292 (3-O-Feruloyl quinic acid), showed the opposite trend (**Figure 3G**). Therefore, SNP1:3064324 in *Si016926m* and SNP1:3176740 in *Si016467m* did affect the synthesis of metabolites in phenolic acids pathway.

### 3.4 QTL analysis of foxtail millet agronomic traits

As the parents of the RIL population, Zhangzagu No.3 and A2 had significant differences not only in plant height (PH) and heading stage (HS), but also in number of grain (NG), length of ear neck (LEN/NL), gross weight (GW), ear length (EL), top second leaf length (TSL) and stem diameter (SD) through evaluation (t-test, p<0.05) (**Supplementary Figures 2A, B**). Further, we evaluated the important agronomic traits of millet throughout the growth period in the millet RIL population for three consecutive years (2016-2018) (**Supplementary Table 1**). The descriptive statistics of each agronomic traits of the population were shown in **Supplementary Table 9**. The agronomic traits of millet RIL populations were quite different, and the average phenotypic coefficient of variation reached 24.05%. By analyzing the correlation of agronomic traits, we tagged two cluster in the correlation heatmap (**Supplementary Figure 2C**). In the first cluster shown in blue box, there were significant positive correlations among 12 agronomic traits, such as ear shape (ES) and anther color (AC). Another cluster shown in green box containing five yield-related traits such as panicle weight (PW) and grain weight (GraW), also showed highly positive correlation.

**Figure 4:**
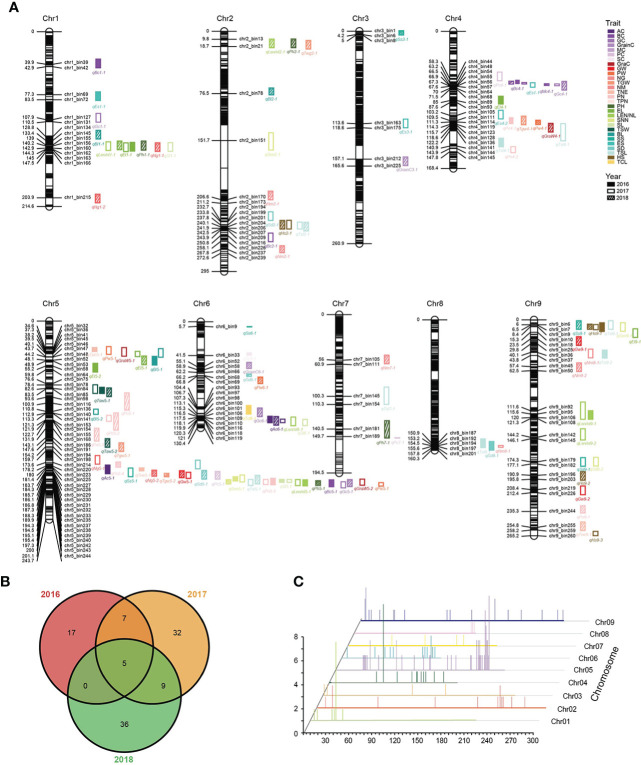
Chromosomal distribution of pQTLs identicfied from agronomic traits. **(A)** Genetic linkage map and pQTLs controlling agronomic traits. **(B)** Venn diagram of pQTLs identified for agronomic traits in three years. **(C)** Distribution characteristics of pQTLs for agronomic traits.

We used the three-year phenotypic data to perform single-environment QTL analysis, and a total of 132 pQTLs (LOD > 2.5) were identified (**Figure 4A**; **Supplementary Table 10**). In the 2016 pQTL results, most of the traits were only mapped to one pQTL, of which the bristle color (BC) mapped a maximum of three pQTLs, while LEN/NL_2017 and PN_2018 can mapped five pQTLs, respectively. This confirmed that agronomic traits were closely linked with environmental factors and affected the pQTL results. Thus, stable pQTLs that had been repeatedly identified in more than one year for the same trait can more accurately reflect genetic characteristics of phenotypes. We identified 21 stable pQTLs across all chromosomes, and more than half of them explained a phenotypic variation of greater than 10% , especially *qBc4-1* with the highest LOD and PVE (**Figure 4B**; **Supplementary Table 11**). Similar to the results of mQTL, the distribution of pQTL on the chromosome was not evenly (**Figure 4C**).

### 3.5 Unearthing of pQTL potential candidate genes

Based on the genome annotation and reported functional genes, we searched for potential candidate genes in the pQTL interval. A total of 39 candidate genes were mined from 25 pQTLs loci with 12 agronomic traits related to crop growth, development and yield formation (**Table 2**). The stable pQTL *qHs9-1* (LOD=4.99-11.45, PVE=10.33-20.85%) for HS located within 0.39-1.06 Mb on chromosome 9, where we found a transcription factor *Si039184m* which encodes a *GHD* protein (**Supplementary Figure 3A, B**). The homologous gene *OsGHD7* of *Si039184m* (Blastp E-Value=8.19E-59, Identity: 49.8%) in rice had been reported to play a major role in regulating the flowering time under long day (LD) conditions by negatively regulating the expression of *EHD1* and *HD3A* (**Supplementary Figure 3C**) (Hori et al., 2013). Another pQTL *qHs9-2* (LOD=3.36, PVE=5.62%) located in the 57.82-58.44 Mb on chromosome 9 was also identified, in this loucs, we found a candidate gene *Si034749m* encoding *EHD4* protein domain (**Supplementary Figures 3A, B**). The function of its homologous gene *OsEHD4* (Blastp E-Value=5.86E-93, Identity: 40.6%) in rice had been proved to be related to flowering as a downstream gene of *GDH7* (Gao et al., 2013). Phylogenetic analysis revealed that both *Si039184m* and *Si034749m* were grouped together with the reported *GDH* genes and *EHD* genes from monocotyledonous cereal crops, respectively, which indicated the consistent function of them (**Supplementary Figure 3D**). At the locus of *qTne9-1* (57.07-57.82 Mb, LOD=2.74, PVE=4.41%) near *qHs9-2*, a yield-related gene *Si035172m* attracted attention. Its homologous gene *OsTE* in rice (Blastp E-Value=0.0, identity: 93.0%) encodes a *Cdh1* protein belonging to a co-activator of APC/C. The APC/C-TE complex has special effect on regulating the lateral branch and tillering of the embryo, which is an important factor to determine the plant type and grain yield (**Supplementary Figures 3E, F, G**) (Netty and Yossi, 2017). The utilization of this segment will be beneficial to cultivate the planting resources of early-maturing and high-yielding millet.

**Table 2:** Candidate genes based on pQTL results.

Trait Name	Year	Main QTL	Chr	LOD	PVE	Interval (Mb)	Locus of millet reference genome	Position (Mb)	Homologous gene
EL	2018	*qEl1-1*	1	3.57	7.27	30.65-32.14	Si017808m,Si017668m	30.79	OsNST1
LEN/NL	2017	*qLen/nl1-1*	1	6.91	17.08	30.65-32.19			
PH	2018	*qPh1-1*	1	2.53	5.83	30.65-32.19			
EL	2016	*qEl1-1*	1	2.98	5.84	31.51-32.41	Si020198m	31.45	OsPAP10c
EL	2017	*qEl1-1*	1	4.58	12.74	31.51-32.41	Si018007m	31.45	OsMPS
SL	2017	*qSl1-1*	1	6.80	12.32	31.51-32.41	Si016559m,Si016509m	31.93	OsARF18
							Si017671m	31.97	OsGA2ox6
HS	2018	*qHs2-1*	2	3.59	4.54	37.25-39.52	Si029202m	38.25	OsGhd7
HS	2017	*qHs2-1*	2	5.40	5.97	37.25-39.52			
ES	2017	*qEs4-1*	4	6.58	8.73	6.46-8.41	Si007994m	5.34	OsGW2
TGW	2017	*qTgw4-1*	4	4.00	8.27	33.43-34.89			
PW	2018	*qPw4-1*	4	2.65	6.02	34.24-34.89			
GraW	2018	*qGraW4-1*	4	3.75	7.05	34.41-37.15	Si008391m	35.64	OsCKX2
PN	2017	*qPn4-2*	4	4.28	9.12	38.14-38.42	Si008326m	39.29	OsPht1
TPN	2016	*qTpn5-1*	5	3.81	6.07	3.34-3.87	Si001947m,Si001632m	3.66	OsYGL8
PW	2017	*qPw5-1*	5	2.65	2.10	3.47-5.25			
GraW	2017	*qGraW5-1*	5	3.54	2.87	3.69-5.47	Si001664m	5.34	OsGSK3
SL	2016	*qSl5-1*	5	22.40	44.77	43.37-43.73	Si002088m	42.28	OsFBP1
SL	2017	*qSl5-1*	5	27.29	45.39	43.37-43.73	Si001831m,Si001110m	42.57	OsPdk1
GraW	2017	*qGraW5-2*	5	18.08	31.93	43.37-44.01	Si000832m	43.04	OsPH1
LEN/NL	2017	*qLen/nl15-1*	5	5.35	11.89	43.37-44.01	Si001573m	43.16	OsGA20ox1
LEN/NL	2016	*qLen/nl15-1*	5	6.59	10.62	43.37-44.01	Si002599m	43.41	OsIG1
PH	2018	*qPh5-1*	5	18.12	34.69	43.37-44.01	Si000027m	44.38	OsDCL1
PN	2016	*qPn5-5*	5	18.63	36.30	43.37-44.01	Si000265m	44.63	OsSPY
PN	2017	*qPn5-5*	5	28.24	53.20	43.37-44.01	Si001955m	44.86	OsPIN5b
PW	2017	*qPw5-2*	5	21.17	36.79	43.83-44.01			
LEN/NL	2017	*qLen/nl16-1*	6	4.07	7.04	33.75-35.10	Si013177m	34.89	OsNACK
SL	2017	*qSl6-1*	6	3.89	3.74	33.97-35.23	Si013875m,Si013885m	34.93	TOGR1
							Si014597m,Si014596m	35.12	SAUR39
							Si013185m	35.19	BC12
							Si014611m	35.25	OsAHP1
							Si013329m,Si013225m	35.56	OsSPY
HS	2016	*qHs9-1*	9	4.99	10.33	0.39-1.06	Si039184m	1.06	OsGhd7
HS	2018	*qHs9-1*	9	7.65	14.19	0.45-1.06	Si034009m	1.09	OsCesA9
HS	2017	*qHs9-1*	9	11.45	20.85	0.45-1.06	Si035876m	1.77	OsRLCK57
EL	2017	*qEl9-1*	9	3.92	8.20	1.06-1.45	Si035170m,Si035172m	57.54	OsCCS52A
TNE	2016	*qTne9-1*	9	2.73	4.41	57.07-57.82	Si039193m	57.58	OsDTH3
HS	2017	*qHs9-3*	9	3.36	5.62	57.82-58.44	Si034749m	58.22	OsEhd4

EL, Ear length; LEN/NL, Length of ear neck; PH, Plant Height; SL, Stem length; HS, Heading stage; ES, Ear shape; TGW, Thousand-grain weight; PW, Panicle weight; GraW, Grain weight; PN, Panicle number; TPN, Total plant number; TNE, Total number of ear.

The yield-related pQTLs were simultaneously mapped in the 33.43 Mb-37.15 Mb interval of chromosome 4, including *qTgw4-1*, *qPn4-1*, *qPw4-1*, *qGraW4-1* (**Supplementary Figure 3H**). The co-location pQTLs of the similly traits was also beneficial for candidate gene mining, and the gene *Si008391m* with annotated function of cytokinin dehydrogenase was excavated in this locus (**Supplementary Figure 3I**). It was found that *OsCKX2* gene (E-Value=6.09E-164, identity: 52.7%) is a homologous gene of *Si008391m* in rice, and *OsCKX2* affected rice yield by regulating the content of cytokinin (**Supplementary Figure 3J**) (Li et al., 2013). This provided important evidence and resources for genetic breeding related to yield in millet.

### 3.6 Exploration of the connection between metabolome and agronomic traits

**Figure 5:**
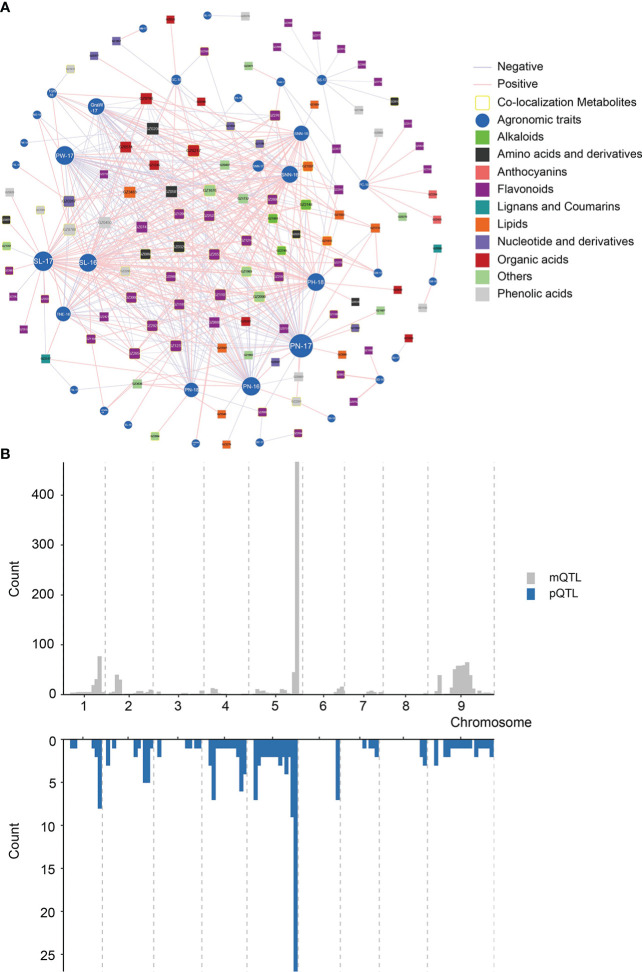
Association network visualization of metabolite-agronomic traits and co-localized anaysis. **(A)** Association analysis of 381 annotated metabolites with agronomic traits. Metabolites and agronomic traits are represented as nodes, and their correlation coefficient values as edges.The absolute values of the Pearson’s correlation coefficient values above the threshold (P < 0.01) are shown. Different colours represent different classes of metabolites. Green rectangles and blue circles are represented as metabolites and agronomic traits, respectively, where the size of the shape represents the number of associations. The level of correlation is indicated as red (positive correlation) or blue (negative correlation). The intensity of the colour indicates the correlation, where a darker colour denotes a stronger correlation. The yellow circles indicate metabolites that are significantly associated with the co-localization of close agronomic traits. **(B)** Co-localization analysis between metabolites and agronomic traits.

In order to explore the relationship between phenotypic characters and metabolome, we focused on co-localized metabolites that were highly correlated with the phenotypic traits. We built a metabolite-agronomic trait association network based on the correlation between 381 annotated metabolites and 63 phenotypes (**Figure 5A**). A total of 498 significant correlations were screened (|r|>0.3, p<0.05), which suggested these metabolites may be involved in the formation of agronomic traits (**Supplementary Table 12**). Among them, most of the metabolites were in the phenylpropane metabolic pathway. Besides, there were 104 mQTLs of annotated metabolites co-located with pQTLs (**Figure 5B**; **Supplementary Table 13**). A total of 48 metabolites were significantly correlated with their corresponding phenotypes, indicating that these genetic loci may affect the phenotype by regulating the content of metabolites. Notably, most of the annotated metabolites with co-localization were in the phenylpropane metabolic pathway, such as GZ0852 (apigenin 7-rutinoside), which was co-located with SD (2018), and the Pearson’s correlation coefficient was 0.98 (**Supplementary Table 12**).

**Figure 6:**
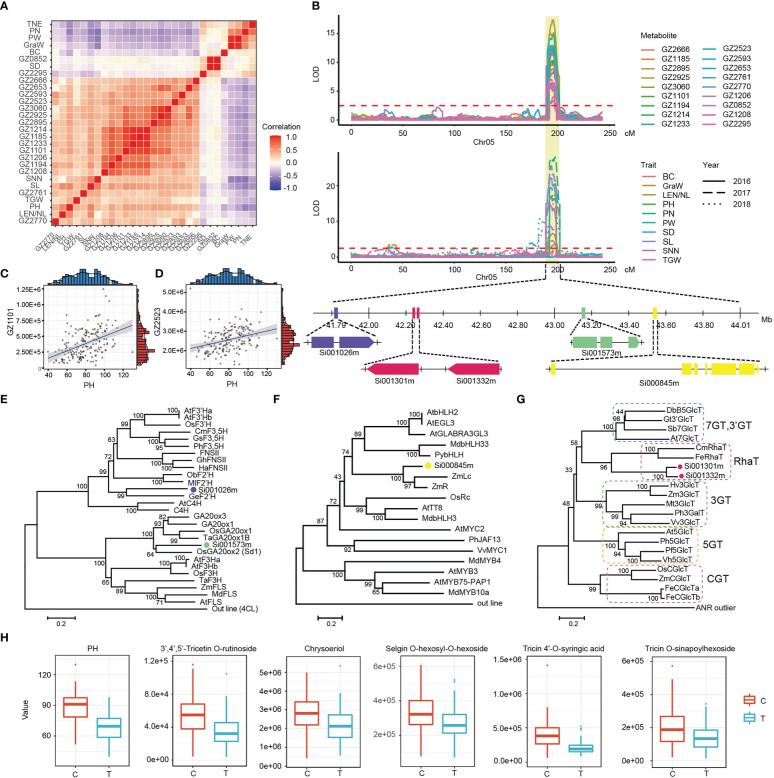
Conjoint analysis of flavonoids and agronomic traits co-localized on chromosome 5. **(A)** Cluster heatmap of correlations between co-localized flavonoids and agronomic traits. Pairwise Pearson’s correlations are shown in a heatmap, flavonoids and agronomic traits are sorted according to correlation-based hierarchical cluster analysis. The level of correlation is indicated by red (positive correlation) and blue (negative correlation). **(B)** LOD curves of QTL mapping of the flavonoids and agronomic traits on chromosome 5. Below the curve is thegene model of candidate genes. The purple box represents the coding sequence of *F2’H*, the light green box represents the coding sequence of *GA20ox*, the pink boxes represent the coding sequence of *UGTs*, the yellow box represents the coding sequence of Lc. **(C, D)** Correlation analysis between two metabolites (GZ1101 and GZ2523) and PH. **(E-G)** An unrooted phylogenetic tree of the candidate genes protein was constructed as described in Methods. Bootstrap values >70% (based on 1000 replications) are indicated at each node (bar: 0.2 amino acid substitutions per site). **(H)** The effect of different alleles on the content of some flavonoids and PH. GZ1206, Chrysoeriol; GZ2593, Syringetin 7-O-hexoside; GZ2666, Selgin O-hexosyl-O-hexoside; GZ2761, 3’,4’,5’-Tricetin O-rutinoside; GZ2653, Tricin -O-(syringyl alcohol) ether 5-O-hexoside; GZ1185, Tricin O-malonyl rhamnoside; GZ1208, Syringetin; GZ2770, Tricin O-sinapoylhexoside; GZ3060, Tricin 4’-O-syringic acid; GZ0852, Apigenin 7-rutinoside; GZ1194, Tricin; GZ1214, Tricin 4’-O-β-guaiacylglycerol; GZ1233, Tricin O-oxalic acid O-coumaroyl shikimic acid; GZ1101, Tricin O-glycerol; GZ2925, Tricin 4’-O-syringyl alcohol; GZ2627, Hydroxymyricetin; GZ2895, Tricin O-phenylformic acid; GZ2954, Tricin -O-syringyl alcohol isomer.

Since the detection of metabolites and the investigation of agronomic traits were carried out in different periods, the high correlation between metabolites and agronomic traits suggested the presence of a genetic relationship. We found that multiple metabolites and agronomic traits were mapped on the same locus 41.33-44.15 Mb of chromosome 5 with high correlation (**Figures 6A, B**). Among them, the metabolites of the flavonoid-lignin synthesis pathway were positively correlated with architecture-related traits, and negatively correlated with yield-related traits (**Figures 6C, D**). Through functional annotation analysis, we found that the *Si001573m* in this interval was the homologous gene of the *Sd1* known as the green revolution gene in Rice (Blastp E-Value=0.0, identity: 82.8%), which can affect plant height by regulating GA synthesis (Sasaki et al., 2002; Spielmeyer et al., 2002; Peng et al., 2021). Phylogenetic analysis showed that *Si001573m* grouped with Gibberellin 20 oxidase of other crops, including *Sd1* (*OsGA20ox2*) in rice (**Figure 6E**). Interstingly, we screened four candidate genes of flavonoid-lignin synthsis pathway nearly the *Si001573m* (**Figure 6B**). Among those, *Si000845m* was annotated as an anthocyanin regulatory Lc protein, its homologous gene in maize has been confirmed to regulate the glycosylation of flavonoids (Blastp E-Value=0.0, identity: 71.0%) (Ludwig et al., 1989; Nesi et al., 2000). Phylogenetic analysis revealed that *Si000845m* was also highly homologous to *Lc* transcription factors in other crops (**Figure 6F**). In addition, *Si001301m* and *Si001332m* encoded glycosyltransferase proteins. Their homologous genes have been reported to play a role in glycosylation modification of flavonoids in strawberry (Blastp E-Value=5.2E-129, identity: 43.8% and Blastp E-Value=5.8E-132, identity: 45.3%, respectively). The catalytic function of *Si001301m* and *Si001332m* for rhamnosyl modification of flavonoids was further determined by phylogenetic analysis (**Figure 6G**) (Lunkenbein et al., 2006). *Si001026m* has been annotated as a flavonoid hydroxyl ligase, and its homologous genes can be used for hydroxylation modification of flavonoid in licorice (Blastp E-Value=4.98E-137, identity: 57.6%). Phylogenetic analysis showed that *Si001026m* was clustered with *F2’H* reported in other species (**Figure 6E**) (Akashi et al., 1998).

There is still a lack of reliable reports or evidences to verify the relationship between metabolites and phenotypes, despite the presence of co-localizations and strong correlation in millet. There were five genes located close together within an ~1.81 Mb region (Chr5: 41.79-43.60 Mb). We speculated that there might be a close linkage between them, causing the non-separation of the same genotype in the hybrid offspring. Therefore, plant height and flavonoid-lignin were highly correlated. In order to confirm this hypothesis, we focus on SNP5: 43169135 near the gene *Si001573m* that formed two different genotypes of C and T in the population. Further analysis revealed that there were significant differences in PH value of millet under the two genotypes (p=3.33E-19), and the content of flavonoid- lignin pathway metabolites such as GZ2761 (3’,4’,5’-Tricetin O-rutinoside) and GZ3060 (Tricin 4’-O-syringic acid) also showed the same trend (**Figure 6H**), which provided important resources for the development of molecular markers related to functional metabolites and agronomic traits at the same time.

## 4 Discussion

### 4.1 Characteristics of foxtail millet metabolome

In this study, we used widely-targeted LC-MS/MS-based metabolic profiling method to detect 3,452 metabolite signals in millet seedling leaves, and elucidated 381 metabolites through standard comparison and chromatography-mass spectrometry data, including primary metabolites and secondary metabolites. Additionally, the coefficient of variation (CV) of primary metabolites was significantly lower than that of the secondary metabolites, which was in line with the results in other cereal crops such as rice, wheat and barley. Interestingly, flavonoids in millet and barley, especially anthocyanins, have the largest CV. However, in wheat, the CV of phenolamide alkaloids was the largest, and the CV of flavonoids and anthocyanins was relatively small (Chen et al., 2013; Chen et al., 2020a; Shi et al., 2020; Zeng et al., 2020).

The correlation between metabolites not only reflect the relationship among metabolites in synthesis pathways and genetic regulation, but also help to predict the structure of unknown metabolites. We observed a significant association between metabolites of the same class, such as flavonoids, phenolic acids, lipids, amino acids and nucleotide. Metabolites in adjacent pathways from different classes still have significant associations, such as flavonoids - anthocyanins, and flavonoids - phenolic acids, which were consistent with the results in wheat and rice (Gong et al., 2013; Shi et al., 2020). Apart from this, some metabolites were not closely connected in metabolic pathways were also highly correlated. For example, the Pearson’s correlation coefficient of GZ2056 (N-(4’-O-glycosyl)-p-coumaroyl agmatine) and GZ1939 (4-Pyridoxic acid O-hexoside) reached 0.66, possibly due to the similar glycosyl structure. Co-localization analysis showed that they were mapped on the same locus of 24.24-28.42 Mb on chromosome 1, indicating that their glycosylation modification may be regulated by the same site (**Supplementary Tables 3, 5**).

In addition to the annotated metabolites, we had also detected a series of unknown signals. The correlation between the unknown metabolites and the annotated metabolites, as well as the co-localization analysis of mQTLs can provide an important resource for future efforts in the identification of unknown metabolites and pathways (Shin et al., 2014; Do et al., 2015). For instance, the unknown metabolite GZ2531 had a high correlation with multiple flavonoid metabolites in the Eriodictyol-Apigenin pathway with the average correlation of 0.82 (**Supplementary Table 14**). They were mapped on the same loucs at 6.30-8.89 Mb on chromosome 9. Moreover, GZ2531 and GZ2536 (Chrysoeriol 8-C-hexoside) had the same Q1, thus they were likely to be isomers of each other (**Supplementary Table 5**).

### 4.2 Genetic features of metabolome and phenotypes in foxtail millet

We used the high-density genetic linkage map of millet to identify 1,049 mQTLs of 922 metabolites. Consistent with the mQTL research on rice, wheat, Arabidopsis, maize and apple, we found that mQTLs were mainly distributed in the form of hot spots on the genome (Khan et al., 2012; Gong et al., 2013; Wen et al., 2015; Knoch et al., 2017; Shi et al., 2020). For example, 1,005 mQTLs identified using metabolites in mature wheat grains were distributed on 68 hotspots, such as chromosomes 1B, 4B and 7A (Shi et al., 2020); mQTLs for rice grains and flag leaves were also distributed on 4 hotspots and 2 hotspots, respectively (Gong et al., 2013). In particular, there was a major hotspot in the 40-44Mb interval on chromosome 5 in millet where most of the mQTLs were located, which was consistent with the research in apple (Khan et al., 2012). Different from other plants, the mQTLs of the same class metabolites in millet were not evenly distributed on chromosomes. For example, the mQTLs of flavonoids were concentrated on two hotspots of chromosome 5 and 9, while the nucleotide mQTLs were only concentrated on the hotspots of the chromosome 5.

We compared the present results with previous mGWAS study based on the genetic mechanism of metabolites in natural variation populations of millet, and found that there were differences in the genetic sites identified for the same metabolite (Wei et al., 2021). There were 49 metabolites with significant association interval, including 59 mQTLs and 350 significant SNPs. From 59 mQTLs, we screened 34 metabolite-related 36 important mQTLs (LOD >5, PVE >10%), and only 12 metabolites could detect similar genetic loci by both study, including 9 unknown metabolites (GZ0797, GZ0814, GZ0894, GZ1055, GZ1201, GZ1272, GZ2255, GZ2256, GZ2300) and 3 annotated metabolites (GZ0755 pentoside Caffeate, GZ2305 (+)-Gallocatechin -hexoside, and GZ2800 Tricin O-vanilloylhexoside). Unexpectedly, we found that different metabolites of the same pathway may be detected at the same genetic locus in different populations. Therefore, mQTL and mGWAS can not only verify the accuracy of metabolite genetic locus mining, but also help improve the metabolite pathway.

A total of 132 pQTLs were identified by single-environment QTL analysis using the phenotypic data of three years. By analyzing the distribution of all the pQTLs, we found 12 hotspots (all of which contained at least 3 pQTLs) on six chromosomes including 81 pQTLs. Among them, hotspots_9 contained the most pQTLs and covered with 15 traits, was located at 42-45 Mb on chromosome 5. In hostspots_9, the range of LOD was 3.63 to 28.24, and the range of PVE was 0.39 to 53.20% (**Supplementary Table 15, 16**). Based on the physical coordinates of the QTL confidence intervals, we compared the present results with previous reports. Four pQTLs (*qPw5-2*, *qPh1-1*, *qPh5-1*, *qTgw5-1*) were overlapped with the genomic regions of *qpw5*, *qph1*, *qph5* and *qtgw5* that were isolated from 439 RIL populations in foxtail millet (Zhang et al., 2017). The physical position of *qPw6-1* was overlapped with that of *qpw6.2* for PW detected in a backbone line Ai88 × Liaogu1 F2 population (Zhi et al., 2021). Above all, the QTL analysis in this study was reliable to further explore the genetic relationship between metabolites and phenotypes.

### 4.3 Advantages of metabolome profiling for phenotypic genetic loci research

Metabolites are the closest phenotypic link in the process from heredity to phenotype (Luo, 2015). Metabolome has striking advantages in analyzing less observable phenotypes, which can clearly reflect small changes in phenotypes from metabolite content levels. Through correlation and co-localization analysis, we identified strong associations between metabolites and agronomic traits in millet. For example, PC and two anthocyanin metabolites GZ2284 (Cyanidin 3-O-malonylhexoside) and GZ2423 (Pelargonidin 3O-malonyl-malonylhexoside) showed a significant positive correlation. GZ2284 and GZ2423 could effectively regulate plant color, which was consistent with the research in Arabidopsis and apple (Jiang et al., 2019; Kim et al., 2019). Studies in rice, sorghum and wheat have shown that the downstream metabolic pathways of phenylpropane, tryptophan and tyrosine of shikimic acid can regulate the yield of cereal crops, showing a significant positive correlation with yield (Chen et al., 2020b). However, we found the yield-traits of millet were negatively correlated to GZ0329 (L-Phenylalanine), GZ0200 (L-(-)-Tyrosine) and flavonoid metabolites (based on Tricin-related metabolites), and positively correlated to unsaturated fatty acids, succinic acid. This may because of the specificity of metabolites accumulation pattern in different species or tissues.

The genetic basis of metabolites and phenotypes is mainly divided into three types. First, functional genes that regulate metabolites are distributed near phenotype-related genes, there may be close linkages between these genes. Selection for agronomic traits during the breeding process will also select genes that regulate metabolites through the free-riding effect (Zhu et al., 2018). Secondly, transcription factors that regulate both phenotypes and metabolic pathways can simultaneously affect specific agronomic traits and metabolites (Pillet et al., 2015). Finally, genes can influence the phenotypes by regulating the content of metabolites that affect the phenotypes (Jiang et al., 2019). At present, the identification technology of trait genetic locus has been gradually mature, but the process of mining the candidate genes remains a huge challenge. Many studies only identified QTL or SNP markers and failed to dig out important candidate genes within the locus. According to further genetic mechanism research, we found that metabolites and phenotypes with strong relevance were located in the same locus, which was consistent with the research in wheat and rice (Gong et al., 2013; Shi et al., 2020). In this study, combined analysis of mQTL and pQTL can effectively help narrow the candidate interval and validate key phenotype-related genes.

### 4.4 Comparison of predictions of agronomic traits

With the progress of various omics technologies, multi-omics data have been used to predict complex agronomic traits. Such application enriches the methods of molecular marker-assisted breeding and brings breakthroughs for the genetic improvement of crops (Xu et al., 2014; Wang et al., 2017; Xu et al., 2017). Due to the strong association between seedling metabolites and important agronomic traits throughout the growth period, we attempted to predict agronomic traits using multi-omics data in this study. Based on least absolute shrinkage and selection operator (LASSO) and BLUP models, genomic (2,202 bins of 33,579 SNPs integrated), metabolomic (3,452 metabolic signals) and multi-omics data (genomic and metabolomic data integration) were used to predict the 63 agronomic traits, respectively. Due to the sparse solution characteristics of the LASSO model, the phenotypes were not completely predicted. The average predictability of different data were 0.60, 0.57, and 0.60, respectively. The BLUP model can complete the prediction of all phenotypes with an average predictability of 0.68, 0.66 and 0.79. The predictability of all phenotypes using the BLUP model and multi-omics data has reached more than 0.5, indicating that this method can be used to predict the phenotype of millet and achieve satisfactory results (**Supplementary Table 17**). Among the phenotypes, PN_2017 showed the highest predictability, with an average of 0.85 under all methods (**Supplementary Table 18**).

**Figure 7:**
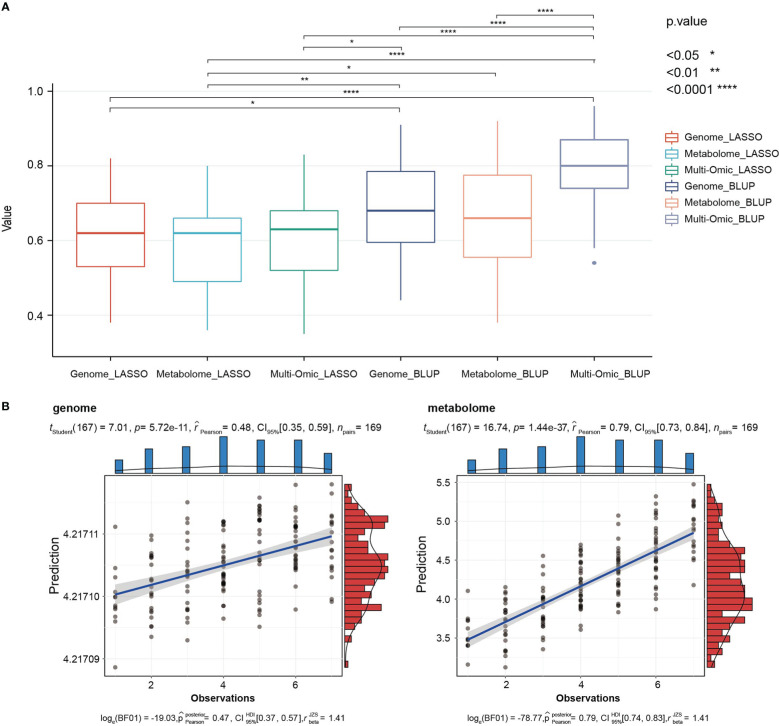
Predictabilities of 63 traits from three omic data (genomic, metabolomic and multi-omics) and two methods (LASSO, BLUP) in the RIL population. **(A)** Box plots compare the prediction results of the two methods for different omics data. In each box-plot, the line in the middle of the box represents the median. **(B)** The predictability of low-heritability phenotypes (TPN_2016) based on BLUP model using genomic (left) and metabolomic (right) data were displayed by correlation scatter plots.

Comparing the prediction results of different omics data by two models, the prediction using BLUP model and multi-omics data was significantly better than other methods. (**Figure 7A** and **Supplementary Table 17**). These results were consistent with the reports in rice and maize (Riedelsheimer et al., 2012; Xu et al., 2016). Based on the correlation analysis of the relationship between the predictability and heritability, we found that the average correlation between predictability of genome participation and heritability was 0.71, 0.69, 0.58, 0.71, respectively, while the correlation between the predictability of phenotype by the metabolome and heritability was smaller, showed 0.36, 0.44, respectively (**Supplementary Table 17**). It showed that the use of genomes for phenotype prediction comparisons relies on the heritability, while metabolomes have advantages in predicting low-heritability phenotypes. For example, the heritability of TPN_2016 was only 0.09, and its predictability based on the BLUP model using genomic and metabolomic data reached 0.48 and 0.79, respectively (**Figure 7B**). Similar results were obtained in rice, metabolomic prediction for YIELD with low heritability was almost twice as efficient as genomic prediction (Xu et al., 2016), and it may be one of the reasons for the better predictability of multi-omics data.

## 5 Conclusion

In this study, LC-MS-based widely targeted metabolic profiling analysis was performed in 179 millet RIL populations. All metabolites and important agronomic traits were performed linkage analysis using high-density genetic linkage maps. A total of 1,049 mQTLs were mapped and distributed in 11 hotspots. We have mined 28 metabolite-related candidate genes from 14 mQTLs by the structure of metabolites and functional annotations. In addition, 136 pQTLs associated with 63 phenotypes were identified by linkage analysis. We found 12 hotspots and 39 candidate genes related to agronomic traits, including *Sd1* which can affect plant height by regulating GA synthesis. Besides, we found that flavonoid-lignin pathway maybe closely related to architecture and yield, and the traits were highly correlated and co-located at chromosome 5. Moreover, we also verified that a combination of genomic and metabolomic for BLUP analysis can more effectively predict plant agronomic traits in millet. In a word, it is of important significance to the study of genetic, metabolic and agronomic traits in millet, as well as molecular breeding involving functional metabolites in crops.

## Data availability statement

The original contributions presented in the study are publicly available. This data can be found here: European Nucleotide Archive, PRJEB56751.

## Author contributions

ZZ and XW designed the research. WW and SL supervised this study. WW, XZ, GaS, GuS, WYZ, JP, DW, XL and YZ participated in the material preparation; WW, FZ, XW, FW, XF and WD carried out the metabolite analyses; PL, KY, YW, SL, WQZ and GF performed the annotation of the metabolites and performed the data analysis. WW and SL discussed the results and wrote the manuscript. All authors contributed to the article and approved the submitted version.
