# Supplementary material for: QTL analysis of important agronomic traits and metabolites in foxtail millet (Setaria italica) by RIL population and widely targeted metabolome
Source: Front Plant Sci. 2023 Jan 10;13:1035906. doi: 10.3389/fpls.2022.1035906 (PMC9872001; doi:10.3389/fpls.2022.1035906)
Supplement: Supplementary file 1 [file Image_1.pdf]

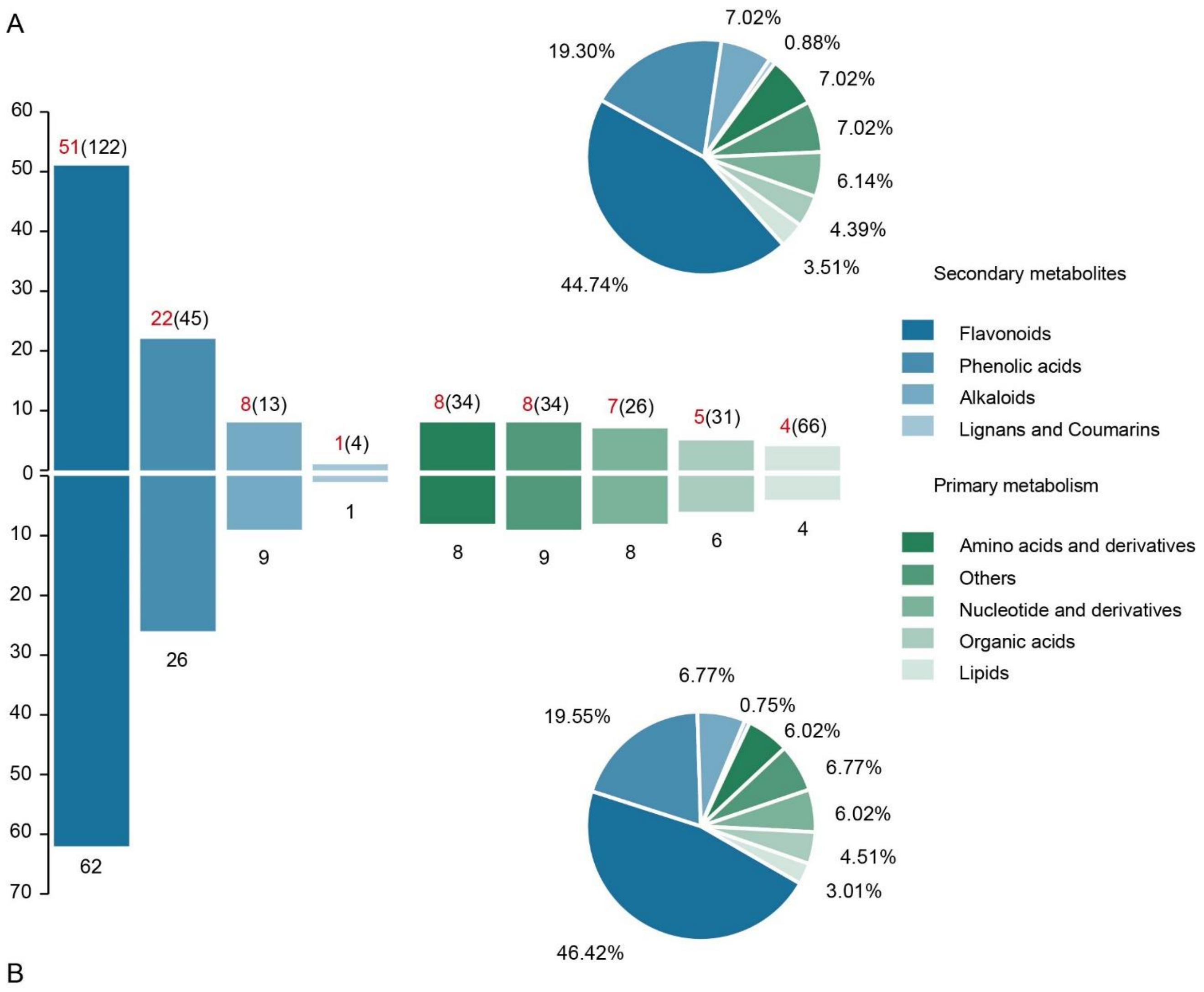

Supplementary Figure 1. Statistical analysis of mQTL for annotated metabolites. **(A)** Distribution of 114 annotated metabolites mapped to mQTL in each Class. The numbers in red represent the number of metabolites with localization, and the numbers in brackets represent the total number of metabolites. **(B)** Distribution of 130 mQTLs of annotated metabolites in different classes.

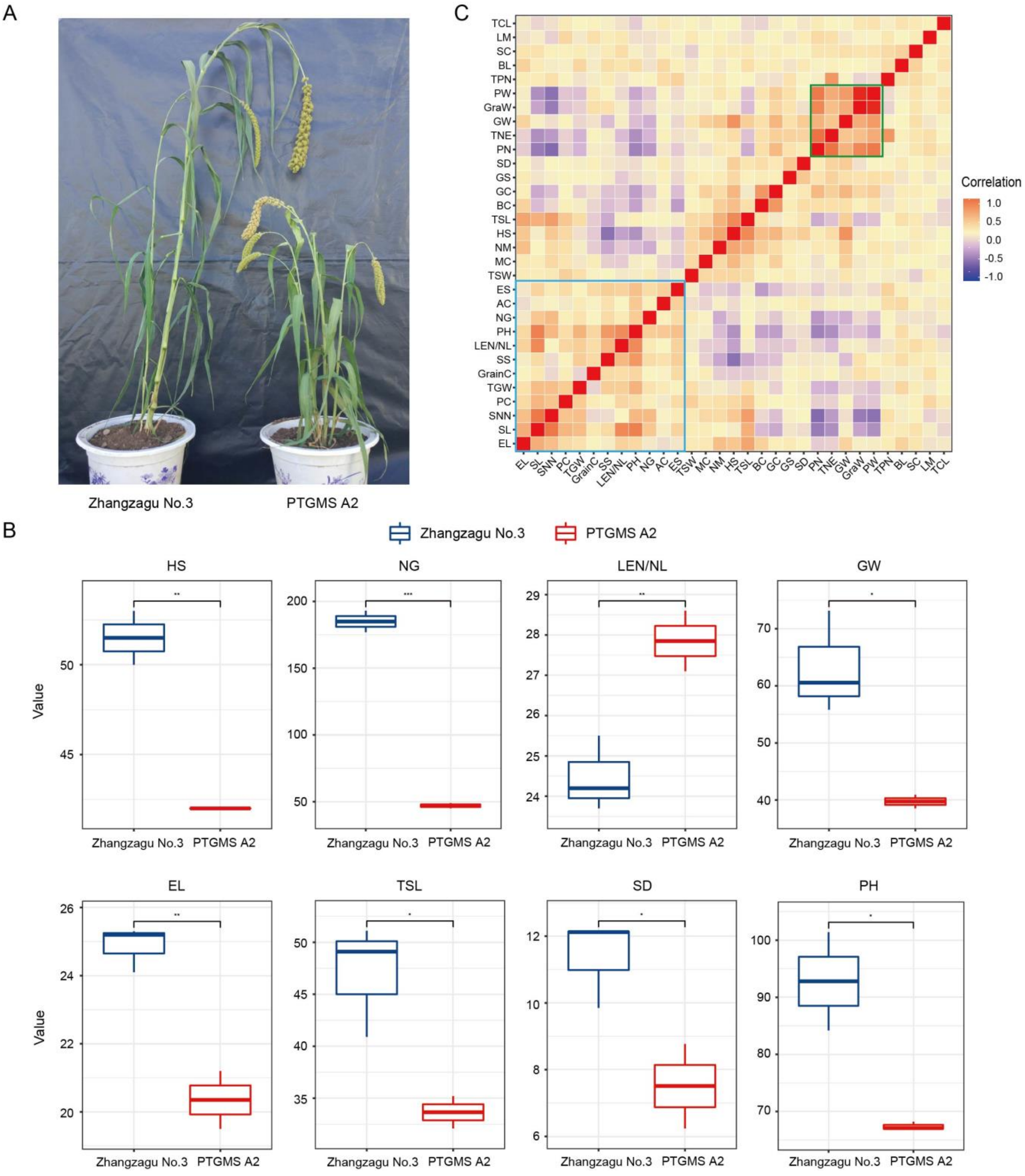

Supplementary Figure 2. Phenotypic differences between two parents of RIL Population and correlation analysis between population agronomic traits. **(A)** Agronomic phenotypes of the two parents Zhangzagu No.3 and PTGMS A2. **(B)** Box diagram of eight agronomic traits for two parents. **(C)** Cluster heatmap of correlations among agronomic traits. Pairwise Pearson's correlations are shown in a heatmap, agronomic traits are sorted according to correlation-based hierarchical cluster analysis.

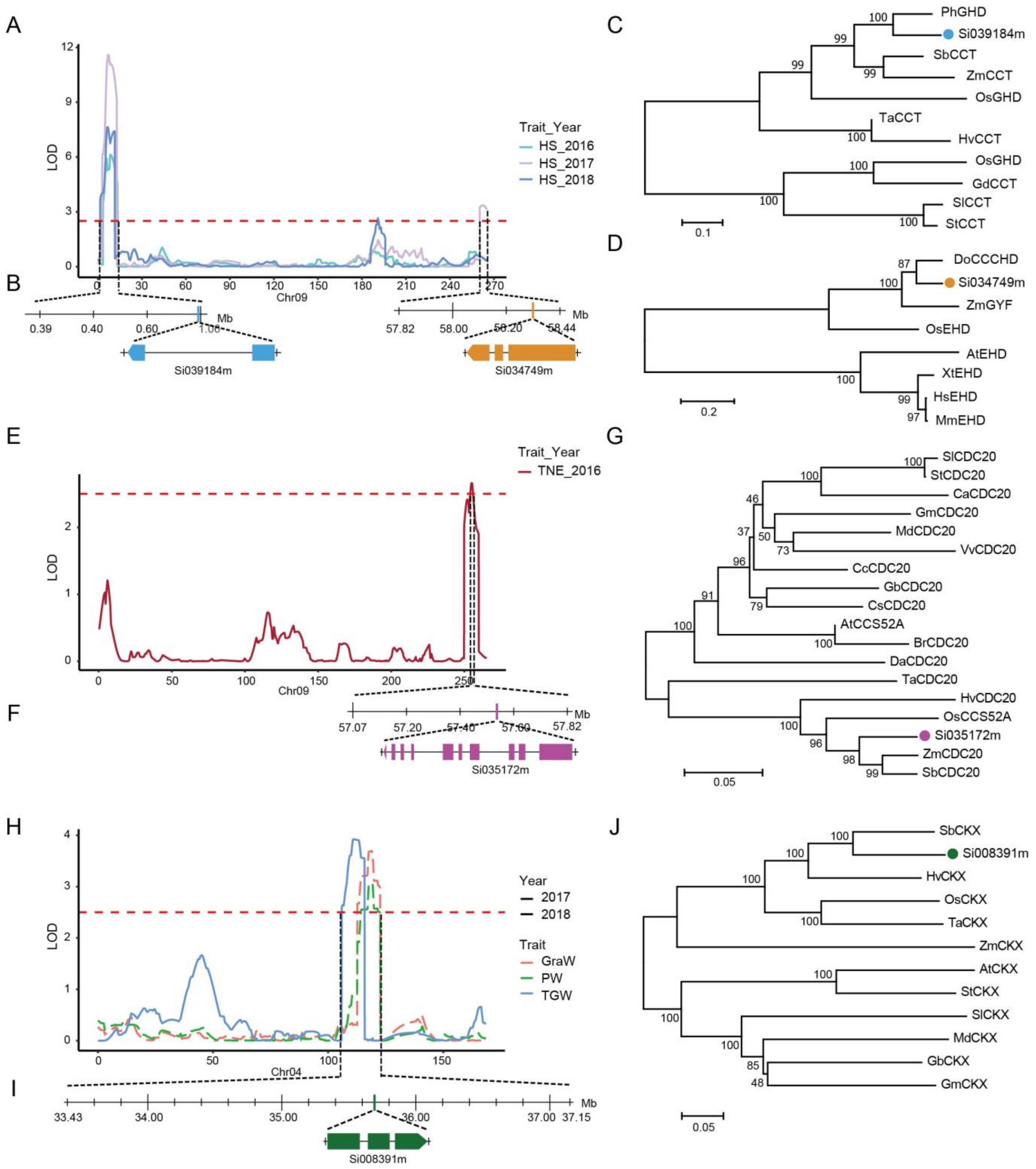

Supplementary Figure 3. Candidate genes for agronomic traits. **(A, E and H)** LOD curves of QTL mapping of HS,TNE and yeild traits (GraW, PW and TGW) in three years and **(B, F and I)** the gene model of candidate genes. **(C, D, G and J)** An unrooted phylogenetic tree of the candidate genes protein was constructed as described in Methods. Bootstrap values >70% (based on 1000 replications) are indicated at each node (bar: 0.2 amino acid substitutions per site).
